# 3D Printed Conductive Nanocellulose Scaffolds for the Differentiation of Human Neuroblastoma Cells

**DOI:** 10.3390/cells9030682

**Published:** 2020-03-11

**Authors:** Matteo Bordoni, Erdem Karabulut, Volodymyr Kuzmenko, Valentina Fantini, Orietta Pansarasa, Cristina Cereda, Paul Gatenholm

**Affiliations:** 1 Genomic and post-Genomic Center, IRCCS Mondino Foundation, Via Mondino 2, 27100 Pavia, Italy; matteo.bordoni@mondino.it (M.B.); orietta.pansarasa@mondino.it (O.P.); 2 3D Bioprinting Center, Chalmers University of Technology, Arvid Wallgrens backe 20, 41346 Göteborg, Sweden; erdkarabulut@gmail.com (E.K.); paul.gatenholm@chalmers.se (P.G.); 3 Wallenberg Wood Science Center, Arvid Wallgrens backe 20, 41346 Göteborg, Sweden; vol.kuzmenko@gmail.com; 4 Department of Microtechnology and Nanoscience, Chalmers University of Technology, Kemivägen 9, 41258 Göteborg, Sweden; 5 Department of Brain and Behavioural Sciences, University of Pavia, Via Forlanini 6, 27100 Pavia, Italy; valentina.fantini@mondino.it; 6 Laboratory of Neurobiology and Neurogenetic, Golgi-Cenci Foundation, Corso S. Martino 10, 20081 Abbiategrasso, Milan, Italy

**Keywords:** 3D bioprinting, cellular models, conductive scaffold, carbon nanotubes, 3D cell cultures

## Abstract

We prepared cellulose nanofibrils-based (CNF), alginate-based and single-walled carbon nanotubes (SWCNT)-based inks for freeform reversible embedding hydrogel (FRESH) 3D bioprinting of conductive scaffolds. The 3D printability of conductive inks was evaluated in terms of their rheological properties. The differentiation of human neuroblastoma cells (SH-SY5Y cell line) was visualized by the confocal microscopy and the scanning electron microscopy techniques. The expression of TUBB3 and Nestin genes was monitored by the RT-qPCR technique. We have demonstrated that the conductive guidelines promote the cell differentiation, regardless of using differentiation factors. It was also shown that the electrical conductivity of the 3D printed scaffolds could be tuned by calcium–induced crosslinking of alginate, and this plays a significant role on neural cell differentiation. Our work provides a protocol for the generation of a realistic in vitro 3D neural model and allows for a better understanding of the pathological mechanisms of neurodegenerative diseases.

## 1. Introduction

The discovery of new clinical treatments or drugs for neurodegenerative diseases (NDDs) and acute traumatic injuries of the neural tissue denotes one of the biggest challenges of modern medicine. For the most common NDDs, such as Alzheimer’s disease (AD), Parkinson’s disease (PD), amyotrophic lateral sclerosis (ALS) and Huntington’s disease (HD), few methods of treatment are available, and they usually provide only symptomatic relief [1,2]. Moreover, the study of the pathophysiology is complicated due to the lack of realistic cellular models of such diseases. For instance, several transgenic animal models helped to understand many pathological pathways [3], but they could not completely recapitulate the human neurodegeneration. The establishment of induced pluripotent stem cells (iPSCs) is considered one of the most important breakthrough technologies of the last decade, representing a very important tool in the NDDs research, because a patient-specific model can be easily created [4,5,6,7]. All the mentioned methods lack the possibility of creating a complex structure that composes human organs, as they generate too simplistic and non-realistic models of human tissues. Thus, there is a need for innovative reliable in vitro models of human NDDs that can help to understand the mechanisms underlying these pathologies.

The development of the 3D bioprinting technology has allowed generation of the realistic models of several human tissues and 3D cell cultures, proposing a connecting bridge with in vivo studies [8]. While several tissues are easily fabricated by the 3D bioprinting, e.g., the bone tissue [9] and cartilage [10], the neural tissue is a more complex tissue, which entails the lack of standardized protocols to obtain a realistic in vitro model of the brain. Moreover, the structure of the neural tissue is very intricate; therefore, great resolution is needed to print it. A bioprinting method called FRESH has been introduced recently as a unique methodology that allows the printing of very complex structures, with an excellent resolution [11]. The FRESH bioprinting relies on printing low-viscosity liquids in a supporting bath of gelatin that can be easily separated from a printed construct. Printed structures are rapidly crosslinked upon printing in a supporting bath that consists of one or more viscous polymer gels. For instance, the gelatin supporting bath has a high viscosity due to its chemical features, allowing it to print scaffolds with high resolution, using low-viscosity liquids [11,12,13].

One of the most significant needs in neural tissue engineering (TE) is the development of the scaffold’s material that is not cytotoxic and supports the neural growth. Moreover, it should mimic the environment in which cells usually live. In 2016, Kuzmenko et al. have prepared nanofibrillated cellulose-based conductive guidelines (NFC) functionalized with carbon nanotubes (CNTs) [14]. It has been demonstrated that the 3D-printed NFC scaffolds have a surface roughness that enhances attachment of SH-SY5Y cells. Moreover, the functionalization with CNTs provides electrical conductivity (about 10^5^ times increase compared with pure nanocellulose), which is prerequisite for cell–cell communication and consequent generation of neural network. The developed bioink takes advantage from three other materials. Specifically, we used alginate, gelatin and Pluronic F-127. Alginate is an optimal biomaterial because of its highly biocompatibility and stiffness. Alginate can be used to model neural tissue, as reported by Fantini and colleagues [15], to implant stem cells or stimulate the metabolism for regenerative medicine [16,17], and to vehiculate molecules on a specific site [18]. Gelatin is often used for its high biocompatibility, but also because it can easily mimic the extracellular matrix [15]. Some groups use it to vehiculate treatments or to enhance healing [18]. Here, we created a gelatin slurry that provided the right stiffness to utilize the FRESH bioprinting method. [11]. Finally, Pluronic F-127 is usually used for the unique thermosensitive property, and Chung and colleagues tried to evaluate its effect in cartilage repair [16]. In our study, we exploited the surfactant feature of Pluronic F-127 that was needed to generate a proper dispersion of CNTs. In the present work, we printed electrically conductive scaffolds, using the FRESH 3D-printing technique. We differentiated SH-SY5Y cells seeded on conductive scaffolds and monitored how the electrical conductivity promotes the establishment of a neural network.

## 2. Materials and Methods

### 2.1. Bioink Preparation

We prepared 1% SWCNTs (P3-SWNT, Carbon Solution, Riverside, CA, USA) water dispersion, using 0.25% of Pluronic F-127 (Sigma-Aldrich, Stockholm, Sweden). Pluronic F-127 was here used as a surfactant, in order to have a good CNTs dispersion. The solution was sonicated at 70 °C for 8 h, followed by sonication at room temperature, overnight. The dispersion was mixed with an aqueous 2% NFC dispersion (Innventia, Stockholm, Sweden) and with 2% alginate (Sigma-Aldrich, Stockholm, Sweden) solution, in order to obtain two conductive bioinks with different CNT content: NFC/CNT/alginate dry weight ratio of 60/20/20 and 70/10/20. We used alginate not for its biological properties, but because we needed a chemical that could be simply crosslinked. We prepared two different types of NFC: an enzymatically degraded noncharged and a carboxymethylated negatively charged NFC. The final mixtures were homogenized, using a SpeedMixer (DAC 150.1 FV-K, FlackTek, Landrum, SC, USA).

### 2.2. FRESH Bioprinting

We slightly modified the protocol proposed by Hinton and colleagues [11]. Briefly, we prepared the gelatin slurry support bath by dissolving 10 g of gelatin (Sigma-Aldrich, Stockholm, Sweden) into 250 mL of 11 mM CaSO_4_ (Sigma-Aldrich, Stockholm, Sweden), at 45 °C. The slurry was stored at 4 °C, overnight. We added 350 mL of 11 mM CaSO_4_ to the gelatin, and, after placing it at −20 °C for 45 min, we blended it for 60 s. We placed 40 mL of blended gelatin into a 50 mL conical tube and centrifuged it for 7 min, at 4100 rpm; the supernatant was then removed, and new gelatin slurry was added, to get a volume of 40 mL. This step was repeated three times, to make all the gelatin blended. The tubes were centrifuged at 225 g for 5 min, at 4 °C, prior to 3D printing; the supernatant was removed, and the slurry was placed into a petri dish. We bioprinted our conductive ink, using Inkredible+ through a 21G needle at a pressure of about 15 kPa. The STL file was created by using Thinkercad (www.thinkercad.com), and it was converted into G-Code, using Slic3r software (https://slic3r.org/). After bioprinting, we placed the Petri dish into an incubator, at 37 °C, for 60 min. The melted gelatin was removed, and after drying, printed scaffolds were sterilized by placing them into 70% ethanol. Finally, the cells were seeded onto the printed scaffolds, for further studies.

### 2.3. SH-SY5Y Cells Cultivation

The human neuroblastoma cell line SH-SY5Y was obtained from Health Protections Agency Culture Collections (HPACC). The cells were cultured in Dulbecco’s modified eagle medium DMEM (Invitrogen, Stockholm, Sweden) with a high glucose level, GlutaMAX™ Supplement (Invitrogen, Stockholm, Sweden) and sodium pyruvate (Invitrogen, Stockholm, Sweden), supplemented with 1% penicillin/streptomycin (P/S) solution and 10% heat-inactivated fetal bovine serum (FBS) (Invitrogen, Sweden). The cells were grown in incubator, under a humidified atmosphere, at 37 °C and 5% CO_2_. The cells were derived from the same batch at passage 19 and were never cultivated beyond passage 25. We tried to evaluate cells viability but we did not achieve it, because the most commonly used methods (i.e., Trypan blue, MTT and Thermo Fisher LIVE/DEAD assay) were not compatible with the scaffold chemical properties.

### 2.4. SH-SY5Y Cells Differentiation

Cell differentiation was performed by following the protocol proposed by Forster et al. [19]. Briefly, cells were cultivated in DMEM (Invitrogen, Stockholm, Sweden) with 1% P/S further supplemented with 10 µM all-trans retinoic acid (Sigma Aldrich, Sweden). The medium was changed with Neurobasal-A medium minus phenol red (Invitrogen, Stockholm, Sweden) with 1% l-glutamine, 1% P/S, 1% N-2 supplement 100× (Invitrogen, Stockholm, Sweden) and human brain derived neurotrophic factor BDNF (Invitrogen, Stockholm, Sweden), at the concentration of 50 ng/mL, after three days. We cultivated cells on these differentiation media for 10 days. The cells were cultivated NFC/alginate (80/20) as negative control and on printed scaffolds with 10% and 20% of CNTs.

### 2.5. SH-SY5Y Cell Imaging

The cell differentiation was monitored by confocal microscopy. Two drops of NucBlue (Invitrogen, Stockholm, Sweden) was added to 1 mL of medium containing living cells. The cells were rinsed three times with PBS 1X and fixed with 4% paraformaldehyde (PFA) in PBS 1X for 15 min. They were then rinsed again and permeabilized with Triton X-100 0.2% in PBS 1X for 15 min. ActinGreen 488 (Invitrogen, Stockholm, Sweden) was added to the permeabilization solution (2 drops for mL), and samples were incubated for 30 min. Finally, samples were rinsed three times with PBS 1X and placed in a microscope slide. Samples were monitored by confocal microscope (LSM 710 NLO, Zeiss, Berlin, Germany).

### 2.6. Scanning Electron Microscopy (SEM)

The samples were rinsed three times in PBS 1X and subsequently fixed for 1 h, at room temperature, in 2% glutaraldehyde dissolved in PBS 1X. Cells were rinsed three times in PBS 1X and dehydrated in a series of increasing ethanol concentration (60%, 70%, 80%, 90% and 100%), with each step lasting 30 min. After samples were completely dried, they were sputter-coated (Fine Coat Ion Sputter JFC-1100, JEOL Ltd., Tokyo, Japan) with 10 nm gold layer, for 80 s, at 10 mA. A LEO Ultra 55 FEG SEM Zeiss scanning electron microscope was operated in secondary electron mode, at an acceleration voltage of 3 kV.

### 2.7. Electrical Conductivity Measurements

The electrical conductivity was measured on crosslinked and non-crosslinked printed scaffolds in dry state before cell seeding. The conductivity measurements were performed by using a two-point probe system (Parameter Analyzer-Keithley 4200-SCS). The working distance between the probes was kept at 3 mm during the measurements.

### 2.8. RT-qPCR

Total RNA content from SH-SY5Y cells was extracted with TRIzol^®^ (Invitrogen, Milan, Italy), following the manufacturer’s instructions. RNA quality and quantity were determined by using NanoDrop spectrophotometer (Invitrogen, Milan, Italy), and 1 μg was reverse transcribed, using the iScriptcDNA synthesis kit (BioRad, Milan, Italy), following the manufacturer’s protocol. PCR amplifications were carried out with the CFX Connect™ Real-Time PCR Detection System (BioRad, Milan, Italy), using SYBR Green Master Mix (BioRad, Milan, Italy). GAPDH gene was used as a housekeeping gene, to normalize values. Primer sequences: NESTIN Fw: 5′-GGA AGA GAA CCT GGG AAA GG-3′, Rv: 5′-GAT TCA GCT CTG CCT CAT CC-3′; TUBB3 Fw: 5′-CAG ATG TTC GAT GCC AAG AA-3′, Rv: 5′-GGG ATC CAC TCC ACG AAG TA-3′; GAPDH Fw: 5′-CAG CAA GAG CAC AAG AGG AAG-3′, and Rv: 5′-CAA CTG TGA GGA GGG GAG ATT-3′.

## 3. Results and Discussion

### 3.1. FRESH Bioprinting

**Figure 1:**
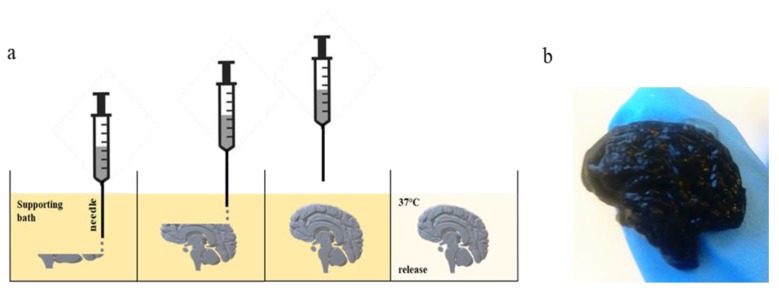
(**a**) Workflow of FRESH bioprinting technique. (**b**) Brain-like scaffold obtained by using cellulose-based bioink printed with Inkredible+.

We bioprinted the cellulose-based bioink, using the Inkredible+ Bioplotter (Cellink AB, Göteborg, Sweden), into a gelatin supporting bath, and then we released the scaffold, placing the Petri dish at 37 °C for 60 min (Figure 1a). As shown in Figure 1b, we FRESH 3D-printed a brain-slice-like construct with a high resolution, in gelatin support. We obtained a scaffold with the realistic shape of a brain, maintaining the intricate surface (Figure 1b). We confirmed the very high resolution, which can be obtained by using the FRESH bioprinting method, which is not useful only for brain-like scaffolds, but also for many other shapes that have fine details. Such a method is particularly useful when it is necessary to have a defined shape for the generation of a 3D model.

### 3.2. Optimization of Scaffold’s Solidification

**Figure 2:**
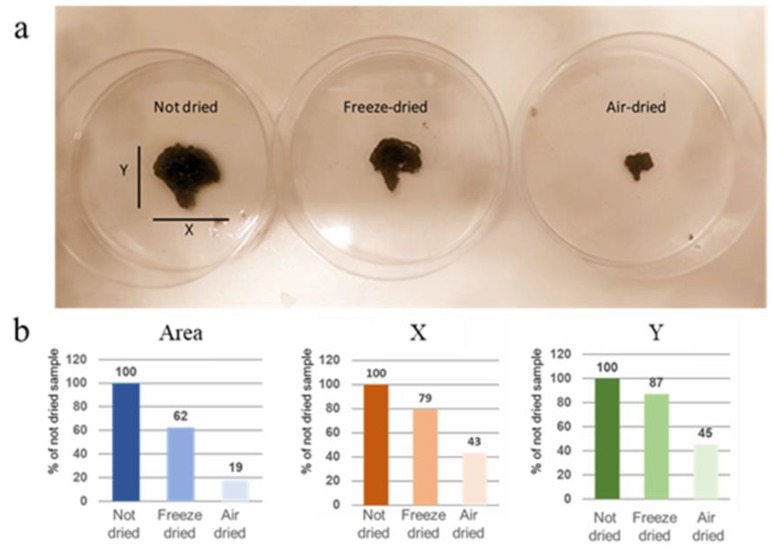
(**a**) Photo of not-dried scaffold compared to freeze-dried and air-dried samples. (**b**) Analysis of X, Y and Area measures, using ImageJ software. Values are % of not-dried sample.

It is important to have a continuous conductive network of CNTs in order to obtain an efficient electrical conductivity. We dried the 3D-printed constructs by freeze- and air-drying. Figure 2a shows the 3D-printed constructs in the gel and dried forms. It is demonstrated that the structures are maintained for both drying methods; however, the air-dried samples have shrunk in all three dimensions upon drying. It has been observed that the freeze-dried constructs keep their dimensions, despite a small portion of shrinkage, and create surface and bulk pores upon freeze-drying. Constructs result in a scaffold more similar to the original one in respect to the scaffold air-dried. Using the ImageJ software, we calculated that X and Y dimensions of air-dried samples are reduced by more than half compared to non-dried samples, while the freeze-dried samples reduced by only 20% (Figure 2b). Moreover, the air-dried samples maintain an area of only 20% of the non-dried samples, while the area of freeze-dried samples is about 60% of the non-dried sample.

### 3.3. The Attachment of SH-SY5Y Cells Is Charge-Dependent

**Figure 3:**
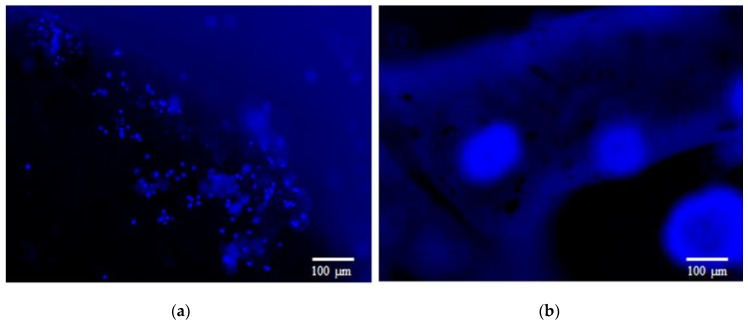
Cells attached to NFC1 scaffold (**a**), while they seem to not attach to the negatively charged NFC8 scaffold (**b**). Cells nuclei were stained blue, using NucBlue. Scale bar: 100 µm.

The cell adhesion occurs because of the many cell-membrane proteins families, such as the so-called integrins, that attach to the extracellular matrix, thanks to protein-to-protein interactions. Therefore, it is critical to fabricate a scaffold that can mimic the extracellular matrix (ECM), allowing the attachment of specific cell types. As shown in previous studies, the cellulose nanofibrils provide a network that mimics the physiological features of the neural ECM, such as cell–material interfacial interaction, optimal stiffness, high surface-to-volume ratio and high porosity [20,21,22]. We seeded the neuroblastoma cells on 3D-printed scaffolds with various compositions, and this provided the different number of charged functional groups. In order to evaluate the effect of charged functional groups on cell attachment, two types of the nanofibrils were evaluated: the enzymatically prepared noncharged NFC, and the negatively charged NFC. The cell-attachment studies have shown that (in Figure 3) the cells have attached only to the noncharged NFC1 surface. The number of cells attached to the negatively charged NFC surface is much lower than the number attached to the enzymatic noncharged NFC surface. This can be attributed to the repulsion between the cells and scaffold surface [23].

### 3.4. Functionalization with CNTs Provides Electrical Conductivity to NFC

Neurons are known to be electrically excitable cells, and several reports suggest that the electrical stimuli in cultivation conditions lead to the improvement of neural characteristics, such as neurites growth and orientation, and communication between cells [24,25]. The NFC is an electrically insulating material; thus, it is necessary to functionalize it with conductive additives. We mixed NFC with SWCNTs that have been classified as biocompatible materials [26] previously.

The conductivity of NFC/SWCNT mixtures with various amounts of CNTs was measured at both crosslinked and non-crosslinked states. It is a well-known phenomenon that the alginate can form a strong and stable network via ionic bonding with Ca^2+^ ions [27] and, thus, successfully crosslink the hydrogel [28,29]. As shown in Table 1, the ionic crosslinking of the constructs has a significant effect on the electrical properties of the 3D scaffolds. The non-crosslinked samples showed rather high electrical conductivity, with a maximum of 2.13 S/cm for the scaffold with 20% of CNTs, which means that the percolation threshold of CNTs is reached [30]. According to previous studies, this level of conductivity is relatively suitable to promote neural cell development [30,31]. On the contrary, the crosslinked scaffold with 10 % of CNTs entirely loses ability to conduct electrons, while the one with 20% of CNTs has very low conductivity. We assume that crosslinked alginate network penetrates through SWCNTs’ conductive chains and obstructs their interconnectivity, leading to the undesired effect of low conductivity.

### 3.5. The Conductive Ink Promotes Differentiation of SH-SY5Y Cells

**Figure 4:**
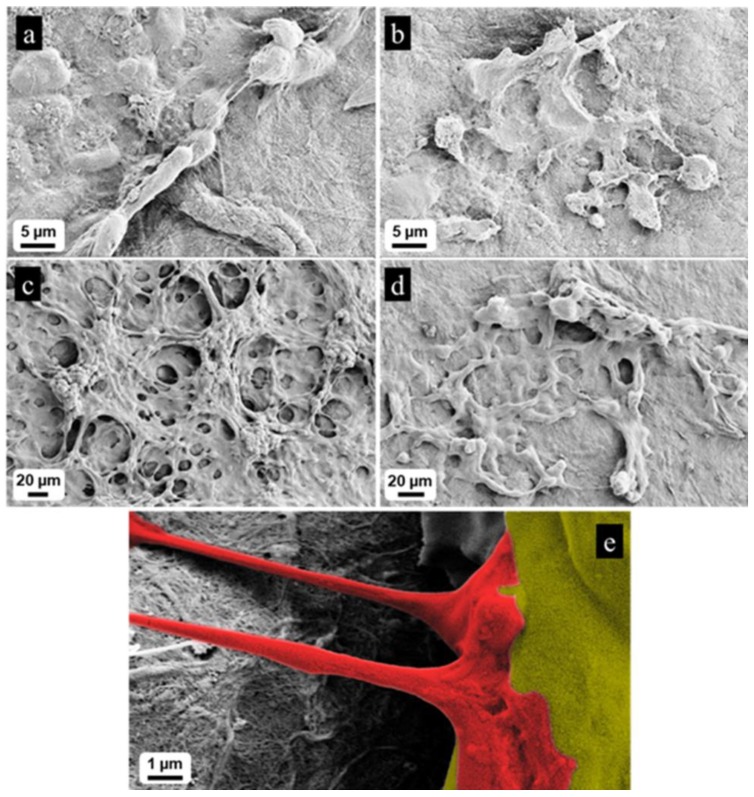
(**a**) Cells cultivated on pure NFC scaffold in presence of differentiation factors started to differentiate in 10 days, but they seem to not generate a neural network. Scale bar: 5 µm. (**b**) Cells cultivated on pure NFC scaffold without differentiation factors did not differentiate. Scale bar: 5 µm. (**c**) Cells cultivated on NFC/10% CNTs with differentiation factors highly differentiated and generated a very complex neural network, with many connections between neurons. Scale bar: 20 µm. (**d**) Cells cultivated on NFC/10% CNTs without differentiation factors differentiated and generated a small neural network. Scale bar: 20 µm. (**e**) Example of synapse found in the sample of cells cultivated in conductive scaffold without differentiation factors. Scale bar: 1 µm.

We have evaluated the influence of the conductive scaffolds on the neural cell differentiation. As shown in the previous study of Kuzmenko et al. [14], the electrically conductive scaffolds that were 3D printed with NFC/CNT ink facilitate the growth and differentiation of SH-SY5Y cells. Our results have shown that the most conductive 20% CNTs scaffolds have no attached living cells, while there were many cells on the 10% CNT constructs. This could be attributed to the cell death or detachment from the 20% CNT-inks, which could have happened as the result of several factors, such as cytotoxity promoted by a too-high concentration of CNTs, as shown in the previous studies [32]. The cells cultivated with differentiation factors on NFC/alginate scaffolds turn into neural cells after 10 days of differentiation process; however, they fail to generate a realistic neural network (Figure 4a). Moreover, when they were not exposed to differentiation medium, they did not differentiate, so they maintained an undifferentiated shape (Figure 4b). On the contrary, when cells were cultivated for 10 days in the presence of differentiation factors on the 10% CNTs scaffolds, they differentiated significantly and were organized into an extended neural network, with many connections between cells (Figure 4c). Surprisingly, when cells were cultivated on 10% CNTs scaffolds without differentiation factors, they managed to show great progress in differentiation after 10 days (Figure 4d) and started to generate a neural network presenting synapses (Figure 4e).

**Figure 5:**
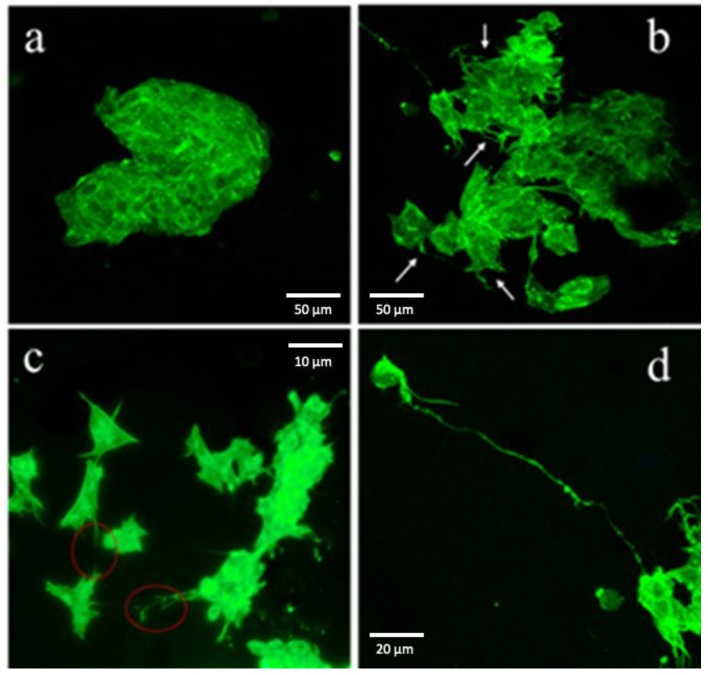
(**a**) Cells cultivated on pure NFC without differentiation factors maintained an immature phenotype, with cell aggregation and without presenting neurites. (**b**) Cells cultivated on conductive scaffold without differentiation factors presented a more neural phenotype, with cells that tend to stay separated and presenting many neurites. (**c**) Interaction between neural cells cultivated on conductive ink. (**d**) Very long neurite of a neural cell cultivated on conductive ink.

The cell differentiation was monitored by the confocal microscopy. The cells were seeded on pure NFC and on 10% CNTs cellulose scaffolds. After 10 days, we fixed and stained cells, using ActinGreen 488. It is shown in Figure 5a that cells cultivated on pure NFC films present a typical undifferentiated cancer-cell shape, with immature phenotype; they have sharp edges and tend to generate colonies. Moreover, they do not have neurites, typical for neural cells. On the contrary, cells on 10% CNTs films differentiated, with many long neurites (white arrows, Figure 5b). It is even possible to monitor neural cells with very long neurites (about 150 µm) that seem to contact other cells (Figure 5c,d).

**Figure 6:**
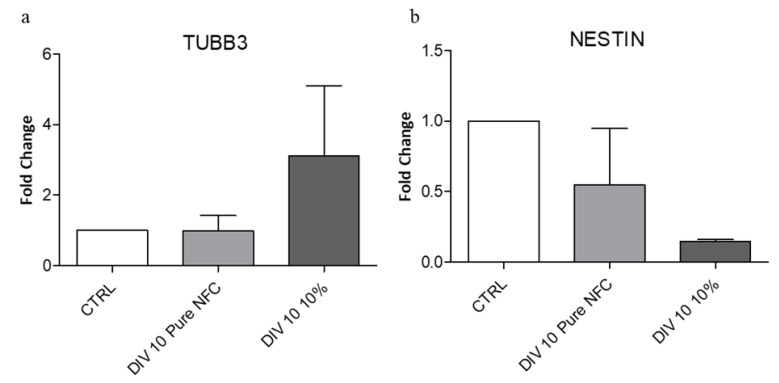
(**a**) TUBB3 expression, a mature neuron marker, increased when cells were cultivated on conductive ink. (**b**) NESTIN expression, an immature neuron marker, decreased in cells cultivated on conductive ink.

Finally, in order to support the morphology data, we analyzed the expression of differentiation markers by RT-qPCR. We investigated TUBB3, a marker that increases in differentiated cells, and Nestin, a marker that decreases during differentiation. In accordance with the results previously reported here, we observed (Figure 6a) an increase in TUBB3 gene expression in the cells cultivated on 10% CNTs scaffold, compared to the cells cultivated on the pure NFC and to the negative control (cells not cultivated). Furthermore, the Nestin gene expression decreased in the cells cultivated on both 3D-printed scaffolds, compared to the negative control, especially on the samples cultivated on the 10% CNTs bioink (Figure 6b). The negligible decrease on the cells cultivated on the pure NFC is probably due to the time of cultivation. The maturation of SH-SY5Y cells into a more neural phenotype could be explained with the ability of the scaffold to transfer current between separated neurons29. The conductive surfaces can promote the cell differentiation significantly and the generation of a neural network without specific differentiation factors. Our data provided evidence that the conductive scaffolds could replace the neural differentiation factors in some cell lines.

## 4. Conclusions

In the present work, we prepared a conductive NFC/CNT-based ink which can be FRESH 3D printed and offer the possibility to prepare conductive scaffolds that promote the differentiation of SH-SY5Y cells. Our work suggests that the NFC-based conductive inks could be utilized for 3D printing of scaffolds for neural cells’ differentiation. It has been demonstrated that 10% wt. CNTs provide enough conductive properties to allow communication between separated neural cells. Electrical conductivity promotes the maturation and the differentiation of SH-SY5Y cells into mature neural cells. Our findings could open up new ways in neural-cell culturing, suggesting that conductive scaffolds fabricated by using the FRESH bioprinting method could help to generate the model of a realistic neural network.

## Figures and Tables

**Table 1:** Analysis of the effect of crosslinking on conductivity and evaluation of conductive properties of scaffolds composed of either 10% or 20% of CNTs. Crosslinking seems to produce loss of conductive effect of CNTs, while non-crosslinked scaffolds have good conductivity (0.12 and 1.9 S/cm, respectively, for 10% and 20% of CNTs).

Name	NFC	CNTs	Alginate	Crosslinking	Conductivity (S/cm)	St. Dev.	*P*-Value
Gel 1	70%	10%	20%	✓	0	-	-
Gel 2	60%	20%	20%	✓	0.007902	0.006475	* <0.026
Gel 3	70%	10%	20%	✘	0.205	0.109473	** <0.0028
Gel 4	60%	20%	20%	✘	2.132	0.571376	**** <0.0001
